# Sotetsuflavone inhibits proliferation and induces apoptosis of A549 cells through ROS-mediated mitochondrial-dependent pathway

**DOI:** 10.1186/s12906-018-2300-z

**Published:** 2018-08-09

**Authors:** Shaohui Wang, Yanlan Hu, Yu Yan, Zhekang Cheng, Tongxiang Liu

**Affiliations:** 1 School of Pharmacy, Minzu University of China, No. 27 Zhongguancun South Street, Haidian District, Beijing, 100081 China; 2 School of Pharmacy, Minzu University of China, Key Laboratory of Ethnomedicine (Minzu University of China), Minority of Education, No. 27 Zhongguancun South Street, Haidian District, Beijing, 100081 China

**Keywords:** Sotetsuflavone, Reactive oxygen species (ROS), Apoptosis, Mitochondria-dependent pathway, Cycle arrest

## Abstract

**Background:**

Sotetsuflavone is isolated from *Cycas revoluta* Thunb., which has biological activity against tumors. However, the anti-proliferative effects of sotetsuflavone on A549 cells and its mechanism are not fully elucidated.

**Methods:**

This study investigated the mechanisms of growth inhibition, cell cycle arrest and apoptosis in non-small cell lung cancer A549 cells induced by sotetsuflavone and evaluated whether sotetsuflavone can be safely utilized by humans as therapeutic agent.

**Results:**

We found that sotetsuflavone had significant antiproliferative activity against A549 cells. At the same time, the reactive oxygen species (ROS) content increased while the mitochondrial membrane potential and the ratio of Bcl-2/Bax decreased. Cleaved caspase-3, cleaved caspase-9, cytochrome C and Bax expression increased, and Cyclin D1, CDK4, cleaved caspase-8 and Bcl-2 expression decreased. Interestingly, we demonstrated that sotetsuflavone could effectively inhibit the G0/G1 cycle progression, and then induce the endogenous apoptosis pathway. Our results show that sotetsuflavone could inhibit the growth of A549 cells by up-regulating intracellular ROS levels and causing the mitochondrial membrane potential to collapse, inducing G0/G1 phase arrest and endogenous apoptosis.

**Conclusions:**

In short, we confirm that sotetsuflavone had an inhibitory effect on A549 cells and discovered that it causes apoptosis of A549 lung cancer cells. Sotetsuflavone may be used as a novel candidate for anti-tumor therapy in patients with lung cancer.

## Background

Lung cancer, also known as Primary Bronchogenic Carcinoma, is a serious threat to human health and quality of life, ranking first among malignant tumors in morbidity and mortality [1–3]. The morbidity and mortality of lung cancer all over the world has increased rapidly within the last 10 years, and more significantly in developed countries [4]. The cancers are mainly divided into small cell lung cancer (SCLC) and non-small cell lung cancer (NSCLC), and NSCLC accounts for 80–85% [5]. The main counter measures are chemotherapy and radiotherapy, targeted therapy, and immune therapy. Although the existing methods of chemotherapy based on Cisplatin can have some effect, the five years survival rate of lung cancer patients is still only about 17%, while it causes a serious economic burden to the patients [6].

Apoptosis or programmed cell death is important for the stability and growth of cells, as well as is controlled at molecular level [7]. It is associated with several diseases, especially cancer [8]. Many anticancer agents mediate their effects on apoptosis induction [9]. Apoptosis is an active and highly ordered process involving a series of enzymes involved in gene regulation. There are three apoptosis signaling pathways: the mitochondrial pathway, the death receptor pathway and the endoplasmic reticulum pathway, in which the mitochondrial pathway is the major one [10]. Mitochondria release cytochrome c and apoptosis inducing factors, thereby activating downstream apoptotic executors [11]. The mitochondrial apoptosis signaling pathway is regulated by the Bcl-2 protein family [11]. The protein family is divided according to different functions, including the inhibitory subfamily (such as Bcl-2, Bcl-x etc.) and the promoter subfamily (such as Bax, Bak, Bad, Bid). The anti-apoptotic members of the Bcl-2 family are usually up-regulated, while pro-apoptotic members are down-regulated in many cancers [12]. The cysteinyl aspartate specific proteinase family (Caspases) is an important factor in the molecular mechanism of apoptosis and occupies a central role in the process of apoptosis, and its members are directly involved in apoptosis initiation, signal transduction and apoptosis. Among them, cysteinyl aspartate specific proteinase-3 (Caspase-3) is the most critical protease in the apoptosis pathway. Activated Caspase-3 can initiate a Caspase cascade, inducing apoptosis after important protein degradation in cells, caspase 8 is a key factor in the death receptor pathway, and caspase 9 is an important factor in mitochondrial pathway [13]. The cell cycle is precisely ordered and regulated with cyclin and cyclin-dependent kinase (CDK) as key elements [14]. Cyclin D1 is an important cell cycle regulatory protein. By combining with cyclin-dependent kinase 4 (CDK 4), phosphorylation and the deactivation of retinoblastoma protein (pRb), it plays an important role in the G1 to S transition in the cell cycle progression [15]. Reactive oxygen species (ROS) play a key role in cell growth in many cellular signaling pathways [16]. Increased ROS in cancer cells is associated with a variety of changes in cell function, such as cell proliferation, migration, differentiation and apoptosis [8].

*Cycas revolute* Thunb. is an evergreen palm woody plant with ornamental, medicinal and edible value. Its main components are double flavonoid compounds, amino acids and sugars. Ancient records report that it is sweet, flat, astringent, and slightly toxic, with fever-reducing and coagulant abilities, dispersing congestion [17]. We first studied the activity of total flavonoids from *Cycas revolute* Thunb. in vivo*,* and found it can regulate the expression of interleukin-2 and interleukin-10 in immune cells and inhibit the growth and metastasis of tumor cells in lewis lung cancer model mice [18]. To tap its medicinal and edible value, and ensure its safety, we isolated the chemical constituents from *Cycas revolute* Thunb. and carried out anti-tumor activity screening. Sotetsuflavone had the strongest inhibitory effect on A549 cells. Thus, in order to clarify the effect of Sotetsuflavone on A549 cells, we studied its potential molecular mechanism, and evaluated whether Sotetsuflavone can be safely utilized by humans as therapeutic agent.

## Methods

### Plant material, chemicals, reagents, and antibodies

**Fig. 1:**
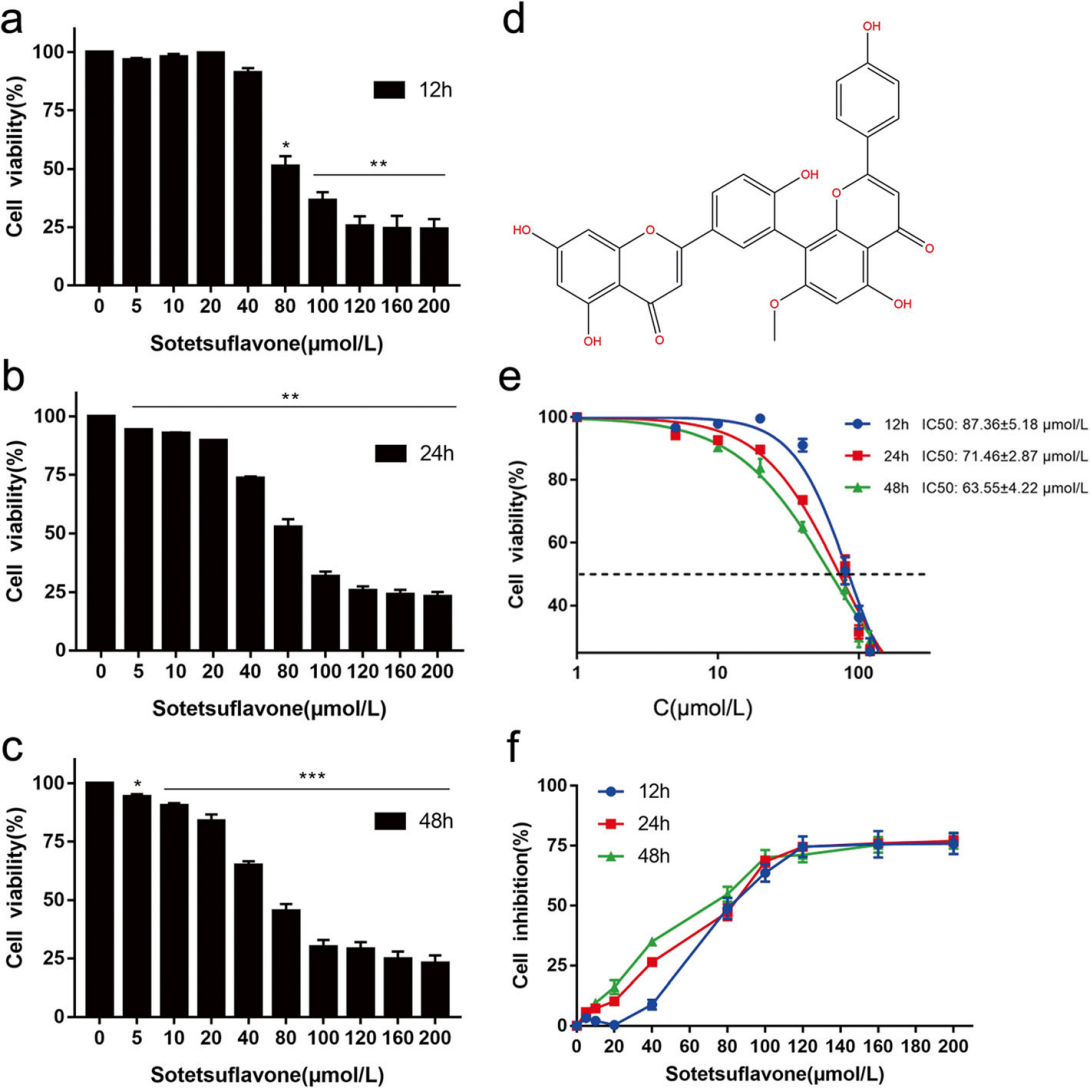
Effects of sotetsuflavone on A549 cells survival. **a**, **b**, **c** show changes of cell viability of A549 cells treated with different concentrations of sotetsuflavone for 12 h, 24 h and 48 h respectively. The viability of A549 cells were significantly different after 12 h, 24 h and 48 h compared with that of control groups (*P* < 0.05, ** *P* < 0.01, *** *P* < 0.001). **d** Molecular structure of sotetsuflavone. **e** The cytotoxicity of sotetsuflavone in A549 cells, there was no significant difference in IC50 values between 24 h and 48 h after drug treatment (*P* > 0.05). **f** The inhibition rate of sotetsuflavone at 12, 24 and 48 h. When the drug concentration was more than 80 μmol/L, the inhibitory effect of the three times gradients was not different (*P* > 0.05). Combined with Fig. 1a, b, c, e, f, the final selection of 24 h as the follow-up experimental treatment time, and the subsequent experimental concentration adjusted to 0, 64, 128 μmol/L. The results from three independent experiments were expressed as mean ± SD compared with the control group, *P < 0.01, **P < 0.01, ***P < 0.001

Sotetsuflavone was isolated from *Cycas Revolute* Thunb. in our laboratory (purity: > 98%, HPLC) (Fig. 1d). The isolation of sotetsuflavone was done using the protocol described by Zhouyan et al. [19]. The leaf of *Cycas Revolute* Thunb. was collected from AnGuo herbal medicine market in HeBei Province of China in May 2015, and was identified by Prof. Tong-Xiang Liu at Minzu University of China. A voucher specimen (No. GRT2015–05) was deposited in the 404 laboratory of Pharmaceutical Research Institute, School of Pharmacy, Minzu University of China, Beijing, China. A549 cells (AS6011), 3-(4,5-Dimethylthiazol-2-yl)-2,5-diphenyltetrazoliunbromide (MTT) assay kit (AS1035), crystalline violet dye (AS1086), Hoechst dye (AS1041) were purchased from Wuhan Aspen Biotechnology Co., Ltd. (Wuhan, China). Dulbecco’s modified eagle medium (DMEM) high glucose medium (SH30022) was purchased from HyClone. (Los Angeles, USA). Cell cycle detection kit (CY2001-O), Annexin-FITC cell apoptosis detection kit (AO2001-02P-G), N-acetyl-L-cysteine (NAC) were obtained from Tianjin three arrows Biotechnology Co., Ltd. (Tianjin, China). JC-1 test kit (C2006), ROS active oxygen kit (S0033), anti-bodies against Cyclin D1, CDK4, Caspase-3, Caspase-9, Caspase-8, cytochrome C, Bcl-2, Bax, and GAPDH were purchased from Beyotime Biotechnology Co., Ltd. (Shanghai, China). DR-200Bs ELISA detection microplate reader was purchased from Wuxi Hiwell Diatek Instruments Co., Ltd. (Wuxi, China). MicroPublisher imaging system (QImaging) was purchased from Shanghai puch Biotechnology Co. Ltd. (Shanghai, China). FACScalibur flow cytometry was obtained from Medical devices Co., Ltd. (BD). (Shanghai, China). CX-21 Ordinary Optical Microscope was purchased from OLYMPUS. (Shanghai, China). All other chemicals made in China were of analytical grade.

### Cell culture

In our previous experiments, we found that sotetsuflavone had a significant growth inhibiting effect on human lung cancer cells (A549) (IC_50_ = 71.12 μmol / L), human colon adenocarcinoma cells (Caco-2) (IC_50_ = 79.70 μmol / L), Human esophageal cancer cells (EC-109) (IC_50_ = 76.68 μmol / L), Human prostate cancer cells (PC-3)(IC_50_ = 106.31 μmol / L) and human hepatoma cells (HepG2) (IC_50_ = 87.14 μmol / L). A549 cells were much more sensitive than the other cell lines. Moreover, sotetsuflavone showed better anti-proliferative activity on A549 than CDDP (Cisplatin) [20]. Based on this results, we used A549 cells to continue the follow-up experiments. A549 cells were cultured in dulbecco’s modified eagle medium (DMEM) medium containing 10% fetal bovine serum, penicillin and streptomycin 100 U/ml, and cultured under constant temperature at 37 °C and 5%CO_2_. Cells were taken during the logarithmic growth phase.

### MTT assay

A549 cells from the logarithmic growth phase were adjusted to 5 × 10^4^ cells/mL, seeded in 96 well plates, 100 μL/well, incubated for 24 h in adherent cells change medium, adding 100 μL in DMEM culture medium with varying concentrations of Sotetsuflavone (0(control), 5, 10, 20, 40, 80, 100, 120, 160, 200 μmol/L). After incubation for 12 h, 24 h, and 48 h, 20 μl of 5 mg/mL MTT working fluid was added to all wells and incubated for 4 h. The supernatant was carefully removed and replaced with 200 μL of dimethyl sulfoxide per well. Plate was then placed on a micro-vibrator for 10 min, and the absorbance at 490 nm was measured [8].

### Cell apoptosis analysis

A549 cells were treated with 0 μmol/L(control), 64 μmol/L, 128 μmol/L sotetsuflavone for 24 h, washed once with precooled PBS and resuspended in 300 μl of a binding buffer diluted with phosphate-buffered saline (PBS). 5 μL Annexin V-FITC was added after incubation for 10 min, then 5 μL PI was added and mixed well, and then incubated for 5 min, detected within one hour by flow cytometry [9].

### Cell cycle analysis

The same method was used to collect A549 cells, washed twice with pre-cooled PBS, and then 70% pre-cooled ethanol was added slowly, gently blowed and resuspended overnight at 4 °C. The cells were collected again before the test, washed twice with pre-cooled PBS, and 500 μL RNase / PI added to resuspend and stained the cells for 20 min, detected within one hour by flow cytometry [14].

### Hoechst 33,258 staining

The cells in the six-well plate were treated as described in the part of cell apoptosis analysis experiment. The cells were washed three times with pre-cooled PBS and then fixed with 4% paraformaldehyde for 30 min, the appropriate amount of Hoechst was added dropwise to the plate after washing with PBS for three times and incubated at room temperature for 15 min, PBS washed three times, then, observed under a fluorescence microscope and photographed [21].

### ROS detection

The cells in in the six-well plate were treated as described in the part of cell apoptosis analysis experiment, collected the cells and added 10 μM DCFH-DA working solution diluted with serum-free liquid. Cells were incubated at 37 °C for 30 min. Reversed the mixture every 3–5 min so that the probe and the cells were in full contact. The cells were subsequently washed 1 to 2 times with serum-free cell culture medium and detected by Flow Cytometry [8].

### Mitochondrial membrane potential detection

The cells in the six-well plate were treated as described above. Cells were collected in 0.5 ml DMEM, and 0.5 mL of JC-1 staining solution was added. The solution was mixed several times and incubated at 37 °C for 20 min. After incubation, cells were centrifuged 5 min, and the supernatant was discarded. The pellet was washed with JC-1 staining buffer (1×) two times, then cells were resuspended in 0.5 mL JC-1 staining buffer (1×) and detected by flow cytometry analysis [9].

### Western blot assay

After the cells were treated with each concentration (0(control), 64, 128 μmol/L), the total protein was extracted with RIPA lysis buffer. The protein samples were subjected to SDS-PAGE and then transferred onto the PVDF membrane. The cells were blocked with Tris-buffered saline tween (TBST) solution containing 5% skimmed milk powder for 1 h. Then the membranes were incubated with primary antibodies of cleaved Caspase-3, cleaved Caspase-9, cleaved Caspase-8, cytochrome C, Bax, Bcl-2, Cyclin D1 and CDK 4 at 4 °C overnight. The membranes were washed with TBST buffer three times and then incubated with a horse radish peroxidase coupled secondary antibodies. Subsequently, the membranes were washed with TBST buffer three times again, and finally detected by the chemiluminescent substrate system and the results were analyzed with the AlphaEase FC software [20].

#### Statistical analysis

The experimental data were processed by SPSS 20.0 statistical software. All experiments were repeated at least three times. The measurement data were expressed as mean ± standard deviation (SD). A one-way ANOVA was used to analyze the variance, when the variance was homogeneous, with a Least Significant Difference, Student-Newman-Keuls test. Conversely, we used the Dunnett T3 test when the variance was not uniform. Differences were considered statistically significant for *P<0.05,**P<0.01,***P<0.001.

## Results

### Growth inhibition of A549 cells by sotetsuflavone

A549 cells treated with 0(control), 5, 10, 20, 40, 80, 100, 120, 160, and 200 μmol/L sotetsuflavone for 12, 24 and 48 h, MTT assay showed a time and dose-dependent inhibition of the growth of A549 cells (Fig. 1a, b, c, f). The IC_50_ values were (87.36 ± 5.18) μmol / L, (71.46 ± 2.87) μmol / L, (63.55 ± 4.22) μmol / L, respectively (Fig. 1e).

### Sotetsuflavone induces apoptosis of A549 cells

**Fig. 2:**
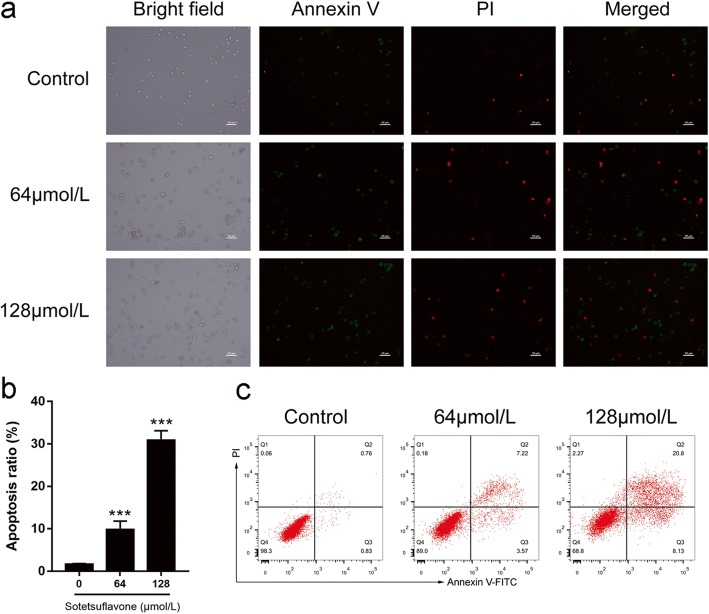
Sotetsuflavone induced apoptosis in A549 cells. **a** Fluorescence observation after treating A549 cells with sotetsuflavone using Annexin V-FITC/PI double staining assay (100 × magnification). **b** Effect of sotetsuflavone on A549 cell apoptosis rate (early and late apoptotic cells) after treating A549 cells with sotetsuflavone. **c** Fluorescence image analysis of apoptotic A549 cells induced with sotetsuflavone by using Annexin V-FITC/PI double staining, where quadrant Q3 are early apoptotic cells, and Q2 are late apoptotic or necrotic cells. The results from three independent experiments were expressed as mean ± SD compared with the control group, ***P < 0.001

**Fig. 3:**
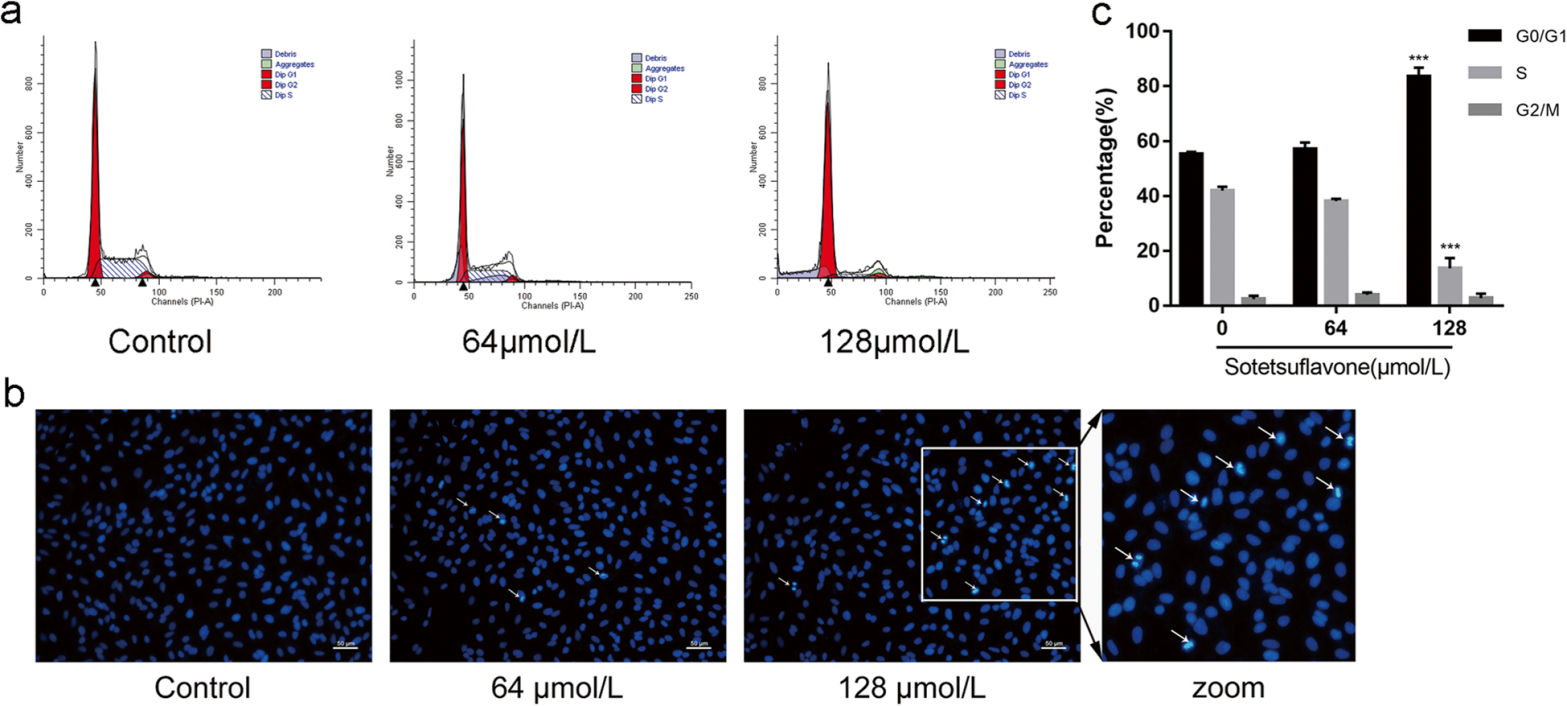
Effect of sotetsuflavone on cell cycle and apoptosis rate in A549 cells. **a** Cell cycle arrest in A549 cells with sotetsuflavone through Flow cytometry. **b** Hoechst 33,258 staining: further evidence of drug induced apoptosis, with the increase of concentration correlated with an increase in the number of dark stained cells, indicating increased, consistent with the flow pattern of Fig. 2c. **c** The table shows the effect of sotetsuflavone on cell cycle arrest in A549 cells. The results from three independent experiments were expressed as mean ± SD compared with the control group, ***P < 0.001

Annexin V-FITC and PI double staining was performed to determine the apoptosis in A549 cells. Untreated cells were primarily Annexin V-FITC and PI-negative, indicating that they were viable and not undergoing apoptosis. After treatment with different concentrations of sotetsuflavone (0 μmol/L(control), 64 μmol/L, 128 μmol/L), intense FITC green in the membrane and PI red in the nucleus were observed (Fig. 2a). Sotetsuflavone induced and accelerated apoptosis of A549 cells, with the increase in drug concentration (Fig. 2b, c). Hoechst 33,258 is a dye that stains the nucleus, which can reflect the number of apoptotic cells, and the even distribution of chromatin, the nuclear-stained uniform blue cells are considered normal but when the nucleus was condensed and fragmented bright blue cells were counted as apoptotic cells. The results of the Hoechst 33,258 staining showed that the A549 cells gradually increased with the increase in of concentration of sotetsuflavone (0 μmol/L(control), 64 μmol/L, 128 μmol/L) (Fig. 3b). And the apoptosis rate increased with increasing drug concentration. We also found that the apoptotic rate of A549 cells is inconsistent with the results of cell viability assay, and this probably because sotetsuflavone promote cell death, not only by inducing cell apoptosis, but also by other types of cell death, such as autophagy. Etc. which requires further research.

### Sotetsuflavone on A549 cell cycle arrest in the G0 / G1 phase

After 24 h, with increasing concentrations of sotetsuflavone (0 μmol/L(control), 64 μmol/L, 128 μmol/L), A549 cells cycle arrest in G0/G1 phase gradually increased (Fig. 3a, c): (55.28 ± 0.94)%,(57.10 ± 2.56)%, and(83.53 ± 3.24)%.

### Sotetsuflavone increases the ROS expression in A549 cells

Reactive oxygen species as an inevitable product of cell metabolism, can play an anti-tumor role by promoting a variety of signaling pathways, such as cell apoptosis, cell necrosis and autophagic cell death. Increased ROS levels can activate ROS-mediated signaling pathways, then causing changes in cell cycle-associated protein levels. The DCFH-DA probe can be hydrolyzed by esterase to form DCFH across the cell membrane, and the reactive oxygen species in the cell can convert the non-fluorescent DCFH into a fluorescent DCF.

**Fig. 4:**
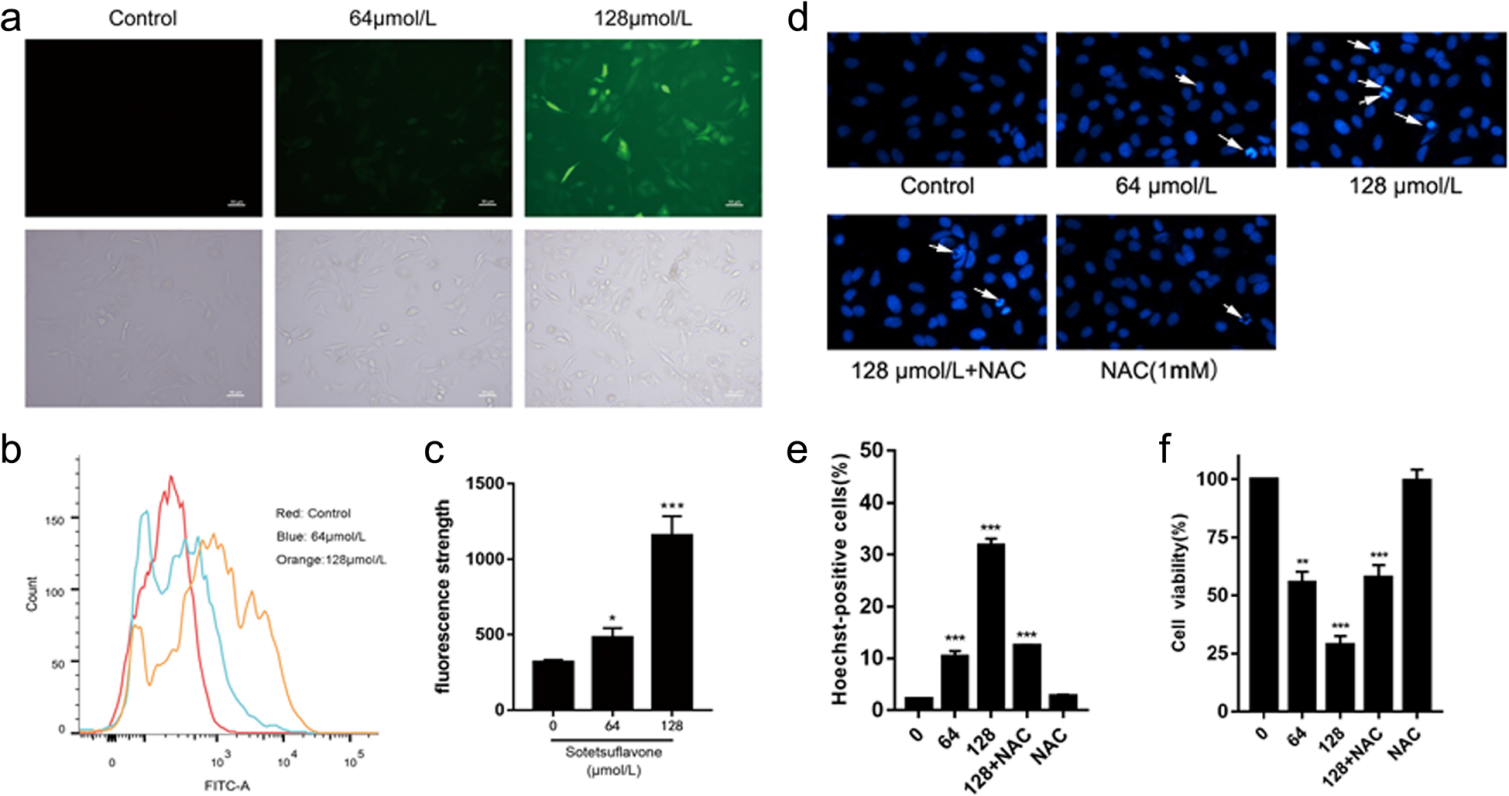
Determination of reactive oxygen species in A549 cells by sotetsuflavone. **a** Fluorescence image analysis of sotetsuflavone inducing apoptosis in A549 cells stained by DCFH-DA(100 × magnification). **b** Average fluorescence intensity of ROS was analyzed by flow cytometry after DCFH-DA staining. **c** Effect of sotetsuflavone on reactive oxygen species in A549 cells. **d**, **e** The experiment was divided into five groups, which were respectively added with 0, 64, 128, 128, 0 μmol/L sotetsuflavone, then, 10 mM NAC solution was added in groups 4 and 5, respectively, thereby became 0(control), 64, 128, 128 + NAC and NAC groups. and the images of fluorescent microscopic analysis sotetsuflavone and NAC respectively inducing apoptosis in A549 cells stained with hoechst 33,258 staining. **f** The experimental group was the same as Fig. 4d, e. and the result showed that inhibitory effect of sotetsuflavone on proliferation of A549 cells. The results from three independent experiments were expressed as mean ± SD compared with the control group, *P < 0.01, ***P < 0.001

The intracellular reactive oxygen species can convert non-fluorescent DCFH into fluorescent DCF [22]. Therefore, the fluorescence intensity of DCF can reflect the level of ROS [23]. It was found that the control group had no green fluorescence and the ROS level was low in the control group, but higher in the treated groups. With increasing drug concentration, the ROS levels in A549 cells increased gradually (Fig. 4a, b, c). The premise of oxidative stress-induced cell apoptosis is the imbalance between oxidation and oxidation resistance in cells. Therefore, we added antioxidant NAC in the experiment. By comparing the changes of each index before and after using NAC, we found that the activity of A549 cells and the number of apoptotic cells were decreased significantly (Fig. 4d, e, f). it was further explained that sotetsuflavone induced apoptosis of A549 cells through the ROS mediated mitochondrial pathway.

### Sotetsuflavone reduces mitochondrial membrane potential of A549 cells

**Fig. 5:**
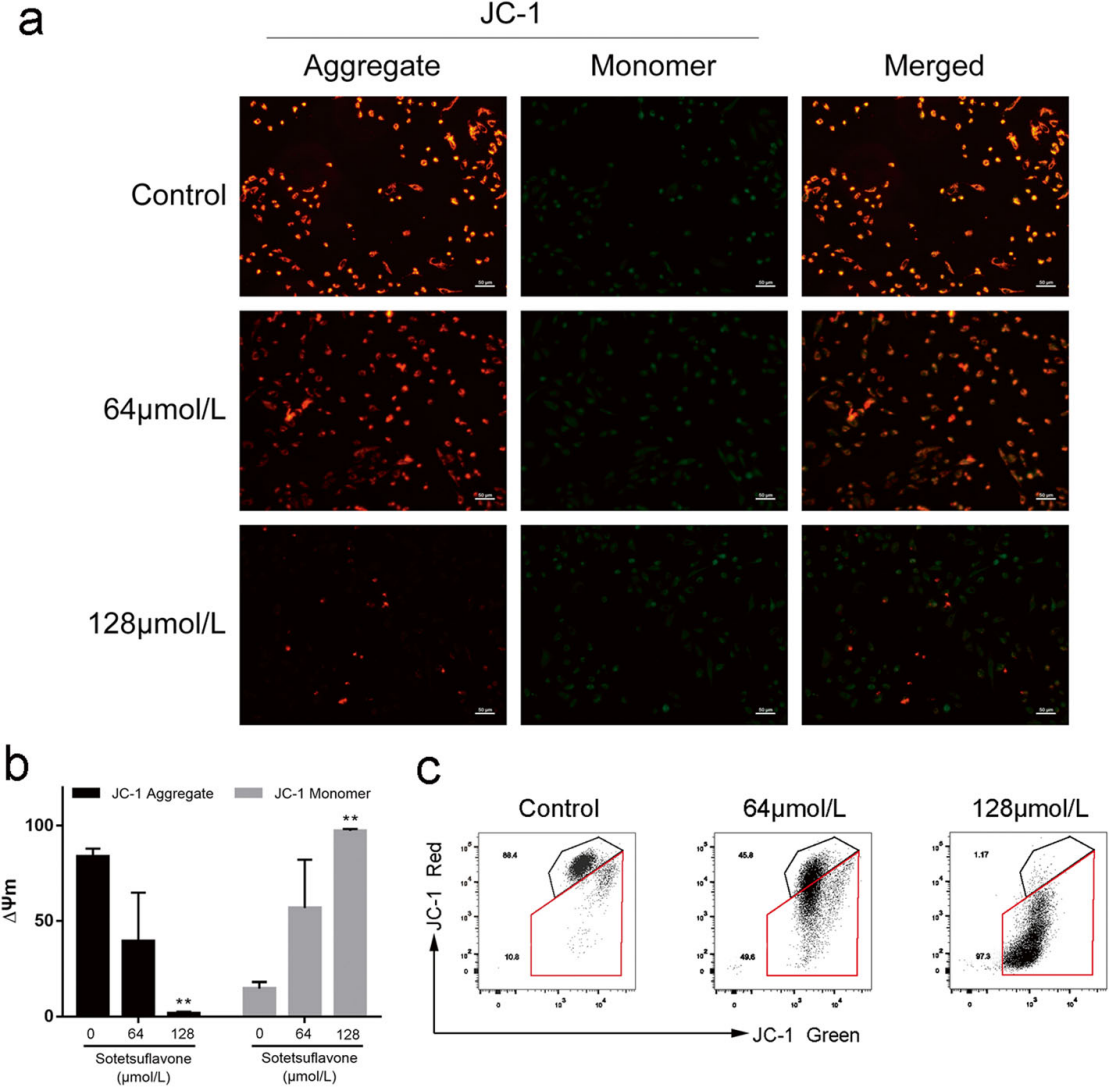
Sotetsuflavone reduces mitochondrial membrane potential of A549 cells. **a** JC-1 fluorescence. **b** Changes of mitochondrial membrane potential after drug treatment. JC-1 Red channel; normal membrane potential cells. JC-1 Green channel; membrane potential collapse of cells. **c** Change of mitochondrial membrane potential after drug treatment. The results from three independent experiments were expressed as mean ± SD compared with the control group, **P < 0.01

After cells are damaged, the membrane potential drops, and JC-1 will appear in the form of green fluorescence. Mitochondrial membrane potential (mitochondrial membrane potential, MMP) collapse is one of the signs of early cell apoptosis. We found that with the increase of sotetsuflavone (0 μmol/L(control), 64 μmol/L, 128 μmol/L), the green fluorescence gradually increased and the mitochondrial membrane potential decreased (Fig. 5a). Consistent with this, flow cytometry results showed that the proportion of cells corresponding to green fluorescence intensity increased from 10.8 to 97.3% with the increase of drug concentration, and the number of cells corresponding to red fluorescence intensity decreased (Fig. 5b, c).

### Sotetsuflavone changes the expression level of apoptosis and cycle-related proteins

**Fig. 6:**
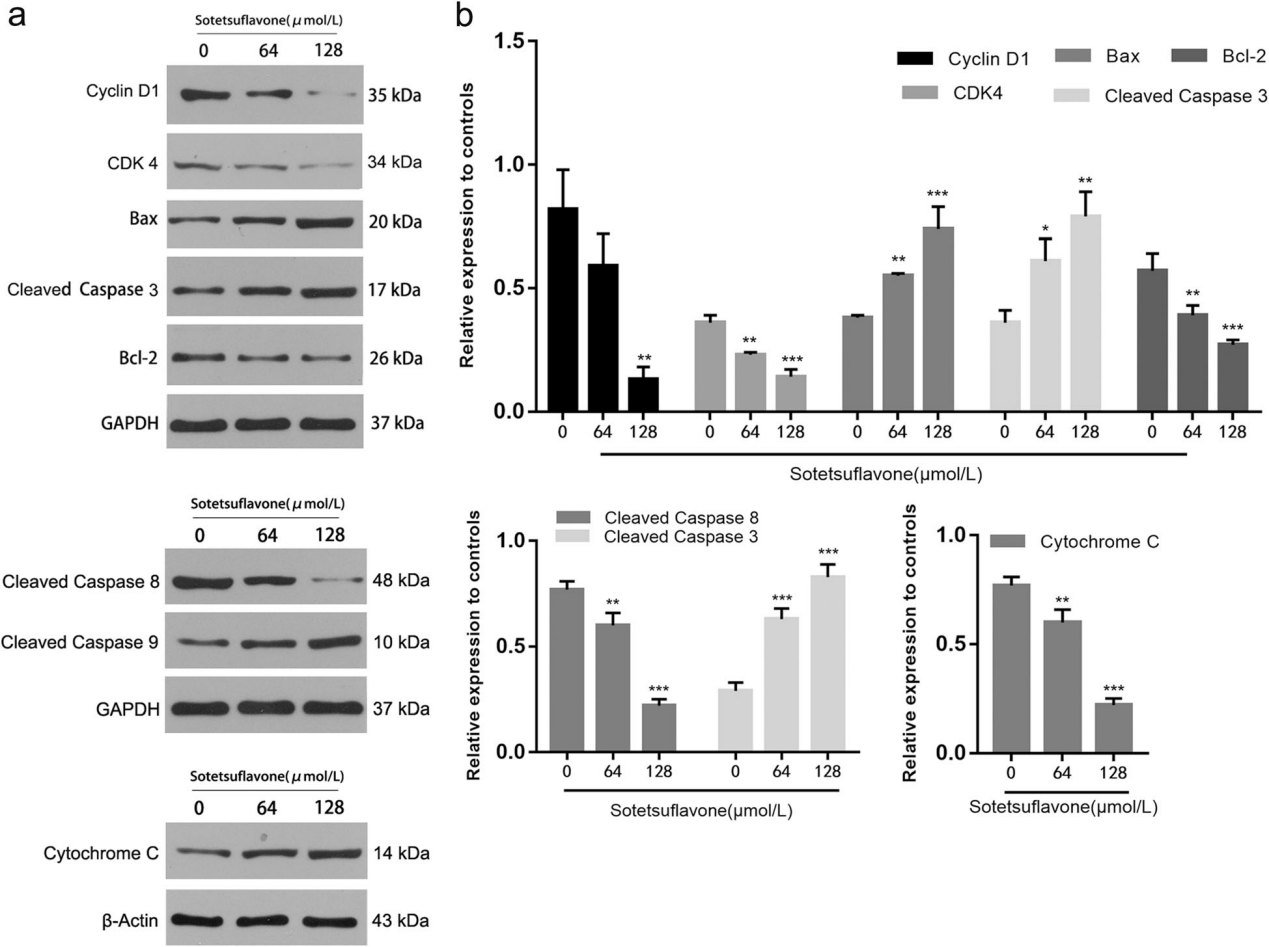
Sotetsuflavone changes the expression level of apoptosis and cycle-related proteins. **a** the expression level of apoptosis and cycle-related proteins modulated with sotetsuflavone for 24 h, GAPDH andβ-actin expression were determined to confirm equal protein loading. **b** The relative expression level of apoptosis and cycle-related proteins. The results from three independent experiments were expressed as mean ± SD compared with the control group, *P < 0.01, **P < 0.01, ***P < 0.001

Apoptosis signaling is regulated by various proteins. Bcl-2, Bax, cleaved-caspase9, cleaved-caspase3, cleaved-caspase 8 and cytochrome C are important apoptosis-related proteins. Compared with the control group (0 μmol/L), sotetsuflavone (64 μmol/L, 128 μmol/L) down-regulated the expression of Bcl-2 protein and up-regulated the expression of Bax protein, which significantly increased Bax/Bcl-2, and it is suggested that sotetsuflavone has a significant induction effect on apoptotic related proteins. The increase of Bax/Bcl-2 can make mitochondrial membrane permeability changes, and cause caspase waterfall activation, which leads to apoptosis. The expression of Cyclin D1 and CDK4 protein was seen to be decreased by detecting cell cycle related proteins, and the progression of G0/G1 phase was inhibited. The expression of cleaved-caspase9, cleaved-caspase3, and cytochrome C were increased, and the expression of cleaved-caspase 8 was decreased, it indicated that sotetsuflavone could not induce apoptosis of A549 cells through death receptor pathway but the mitochondrial pathway (Fig. 6a, b).

## Discussion

Traditional Chinese medicines are convenient, inexpensive, and generally exhibit low side effects. More importantly, traditional medicines can relieve clinical symptoms, and improve the quality of life. Some may even be effective in treating cancer, and here we explore one such possibility.

Apoptosis is an important self-regulating mechanism for multicellular organisms to maintain a stable internal environment. Inducing apoptosis has become a common methodology in tumor therapy and an important index to evaluate the efficacy of anticancer drugs [24, 25]. We showed that sotetsuflavone inhibited the proliferation of A549 cells and showed a dose and time-dependent manner markedly (Fig. 1f). A flow cytometry assay showed that treatment with sotetsuflavone for 24 h resulted apoptosis in A549 cells. Moreover, the proportion of apoptotic cells was significantly increased with elevating levels of drug concentrations (Figs. 2 and 3b). In addition, the study found that anti-apoptosis protein of Bcl-2 expression was down-regulated. On the contrary, the pro-apoptosis protein of Bax was up-regulated (Fig. 6). The Bcl-2 protein family is a special family, some members promote apoptosis, such as Bad, Bid, Bax, and some members block apoptosis, such as Bcl-2, Bcl-w. Bcl-2 can inhibit cytochrome C release from the mitochondria into the cytoplasm, thereby inhibiting apoptosis [26]. Bcl-2 protein is an endometrial protein, mainly localized in mitochondria, endoplasmic reticulum membrane and the nuclear membrane, and is present in a variety of tumor cells. It can increase the mitochondrial membrane potential, inhibit the release of calcium ions, prevent the activation of endonuclease, and then play an anti-apoptotic effect. Bax protein can directly activates the death effect factor caspase or changes the permeability of cell membrane, causing cytochrome C to release ions and small molecules through the cell membrane, thereby promoting cell apoptosis. The Bcl-2/Bax change can regulate apoptosis, when Bcl-2 is dominant, cell have anti-apoptotic effects, Conversely, when Bax is overexpressed, cells are prone to apoptosis [27].

The decline of mitochondrial membrane potential is an early phenomenon of apoptosis. The opening of mitochondrial permeability transition pore (MPTP) decreases mitochondrial membrane potential, and sotetsuflavone reduces the mitochondrial membrane potential of A549 cells in a concentration dependent manner (Fig. 5). Caspase-3 is the most critical enzyme in the pathways of apoptosis. The mitochondrial-dependent pathway of apoptosis is activated as a result of intracellular stress or damage, which engages the Bcl-2 family of pro-apoptosis proteins, including Bcl-2, Bak and Bax [28]. Western blot results showed that the proportion of Bcl-2/Bax protein expression was decreased while Cleaved-Caspase 3 was increased significantly (Fig. 6a). It demonstrated that sotetsuflavone induced apoptosis in A549 cells through mitochondrial-dependent pathway.

Studies have shown that increased intracellular ROS can cause cell membrane potential collapse and thus block the cell cycle, ROS entering the cytoplasm can re-enter the cell nucleus to cause DNA damage, further stimulate endogenous pathway apoptosis, and ultimately lead to cell death [29, 30]. ROS is a key molecule in apoptosis, and Bcl-2 plays a role in the relationship between ROS and apoptosis, regulating and maintaining the activity of intracellular antioxidants. Intracellular ROS produced in apoptotic cells is also associated with changes in Bax protein expressions, generation of ROS in the cells by causing increased expression of Bax and decreased Bcl-2 are considered as indicators of activation of mitochondrial cell death pathway [31]. ROS content increased after 24 h of drug action (Fig. 4a). to confirm whether induction of apoptosis on A549 cells was via ROS-mediated mitochondria-dependent pathway or not, we added antioxidant NAC in the experiment, by detecting the cell viability and apoptosis ratio, we found that after the treatment of NAC, the activity of A549 cells and the number of apoptotic cells were decreased significantly (Fig. 4d, e, f). It is indicated that sotetsuflavone induced A549 cells to apoptosis through the ROS-mediated mitochondrial pathway. The accumulation of ROS disrupts the redox control of cell cycle progression via cell cycle regulatory proteins such as Cyclins and CDKs, leading to aberrant cell proliferation and apoptosis [32]. Cyclin D1 and CDK 4 play an important role in the regulation of cell cycle progression. The expression levels of Cyclin D1 and CDK 4 were significantly reduced by western blotting (Fig. 6a). Flow Cytometry assay showed that the number of apoptotic cells in G0/G1 phase increased with the increase of drug concentration (Fig. 3a, c), which indicated that sotetsuflavone could block A549 cells in G0/G1 phase.

## Conclusions

**Fig. 7:**
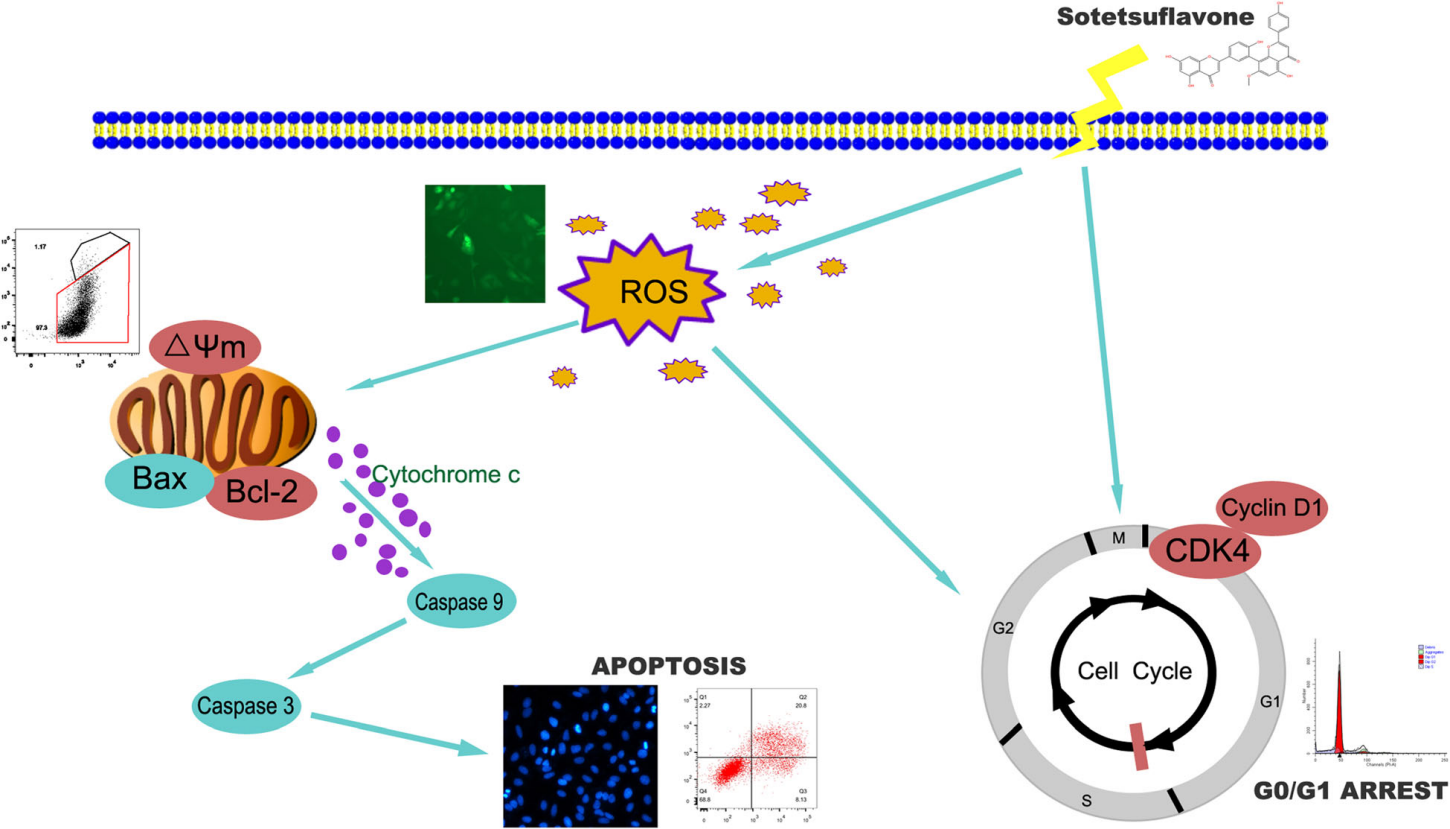
Hypothetical mechanism of sotetsuflavone induced apoptosis of A549 cells

In summary, sotetsuflavone can inhibit the proliferation of A549 cells. The cell cycle is mainly blocked in the G0/G1 phase by increasing intracellular ROS levels, and the enhanced ROS generation resulted in increased oxidative stress, thereby Bcl-2/Bax reduction resulted in an increase in Cleaved-caspase3 expression, subsequently, inducing A549 cells apoptosis (Fig. 7). Taken together, sotetsuflavone induced apoptosis on A549 cells via ROS-mediated mitochondria-dependent pathway. In conclusion, sotetsuflavone may be used as a novel candidate for anti-tumor therapy in patients with lung cancer.

## Abbreviations

Bax: Bcl-2 Associated X Protein
Bcl-2: B cell lymphoma 2
Caspase-3: Cysteinyl aspartate specific proteinase-3
CDK 4: Cyclin-dependent kinase 4
DCFH-DA: 2-(2,7-dichloro-3,6-diacetyloxy-9H-xanthen-9-yl)-benzoic acid
DMEM: Dulbecco’s modified eagle medium
GAPDH: Glyceraldehyde-3-phosphate dehydrogenase
MTT: 3-(4,5-dimethyl-2-thiazolyl)-2,5-diphenyl-2-H-tetrazolium bromide
NAC: N-acetyl-L-cysteine
NSCLC: Non-small cell lung cancer
PBS: Phosphate-buffered saline
ROS: Reactive oxygen species
